# Supplementary material for: Eruca sativa Meal against Diabetic Neuropathic Pain: An H2S-Mediated Effect of Glucoerucin
Source: Molecules. 2019 Aug 19;24(16):3006. doi: 10.3390/molecules24163006 (PMC6721019; doi:10.3390/molecules24163006)
Supplement: Supplementary file 1 [file molecules-24-03006-s001.pdf]

molecules

Eruca sativa Meal against Diabetic Neuropathic Pain: An H_2S-Mediated Effect of Glucoerucin

Elena Lucarini [1], Eleonora Pagnotta [2], Laura Micheli [1], Carmen Parisio [1], Lara Testai [3,4,5], Alma Martelli [3,4,5], Vincenzo Calderone [3,4,5], Roberto Matteo [2], Luca Lazzeri [2], Lorenzo Di Cesare Mannelli [1,*] and Carla Ghelardini [1].

1 Department of Neuroscience, Psychology, Drug Research and Child Health—NEUROFARBA—Pharmacology and Toxicology Section, University of Florence, Florence 50139, Italy
2 CREA-Council for Agricultural Research and Economics, Research Centre for Cereal and Industrial Crops, Bologna 40128, Italy
3 Department of Pharmacy, University of Pisa, Pisa 56126, Italy
4 Interdepartmental Research Centre "Nutraceuticals and Food for Health (NUTRAFOOD)", University of Pisa, Pisa 56126, Italy
5 Interdepartmental Research Centre of Ageing Biology and Pathology, University of Pisa, Pisa 56126, Italy.
* Correspondence: lorenzo.mannelli@unifi.it

Supplementary Materials

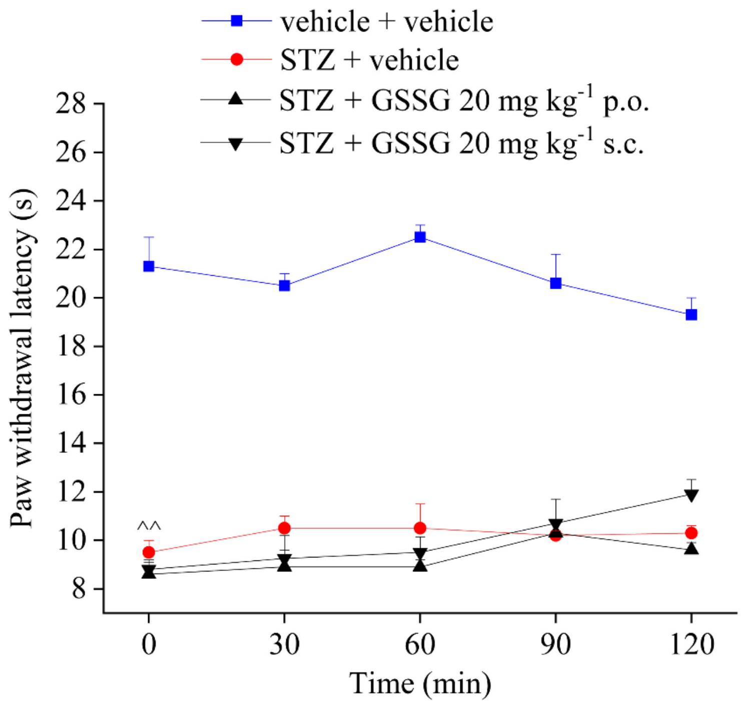

Figure S1. Effect of acute administration of GSSG on STZ-induced neuropathic pain. The response to a mechanical and stimulus was evaluated by measuring the latency to pain-related behavior (withdrawal of the paw). GSSG (20 mg kg-1) was orally and subcutaneous administered in STZ-treated animals and the tests were performed 30, 60, 90, and 120 min after the injection. ^^ $p < 0.01$ versus vehicle + vehicle-treated mice.
